# Advancement in the Development of Therapeutics Against Zika Virus Infection

**DOI:** 10.3389/fcimb.2022.946957

**Published:** 2022-07-08

**Authors:** Kangchen Li, Qianting Ji, Shibo Jiang, Naru Zhang

**Affiliations:** ^1^ Department of Clinical Medicine, School of Medicine, Zhejiang University City College, Hangzhou, China; ^2^ Key Laboratory of Medical Molecular Virology of Ministry of Education (MOE), National Health Commission (NHC) and Chinese Academy of Medical Sciences (CAMS), School of Basic Medical Sciences and Shanghai Institute of Infectious Disease and Biosecurity, Fudan University, Shanghai, China

**Keywords:** Zika virus, pathogenesis, therapeutics, NS2B–NS3 protease inhibitors, NS3 protease inhibitors, NS5 protease inhibitors, E protein inhibitors, host protein inhibitors

## Abstract

Zika virus (ZIKV), a re-emerging arbovirus, causes teratogenic effects on the fetus and normal nerve functions, resulting in harmful autoimmune responses, which call for the development of therapeutics against ZIKV infection. In this review, we introduce the pathogenesis of ZIKV infection and summarize the advancement in the development of therapeutics against ZIKV infection. It provides guidance for the development of effective therapeutics against ZIKV infection.

## Introduction

Zika virus (ZIKV) is a small envelope positive-strand RNA flavivirus belonging to the *Flaviviridae* family (Lin et al., 2018). The genome-encoded polyprotein can be cleaved into three structural proteins [capsid (C), pre-membrane (prM), and envelope (E)] and seven non-structural proteins (NS1, NS2A, NS2B, NS3, NS4A, NS4B, and NS5) (Fontes-Garfias et al., 2017). Transmission routes include arthropod vectors (e.g., *Aedes aegypti*), *via* sexual tranlsmission as well as intrauterine (perinatal) and blood-related routes (Song et al., 2017). ZIKV was also found to be present in breast milk (Dupont-Rouzeyrol et al., 2016; Sotelo et al., 2017). When ZIKV attacks pregnant women, it easily reproduces in the placental tissue and seriously affects the fetal central nervous system and immune system, causing congenital Zika syndrome (CZS) in infants (Driggers et al., 2016; Noronha et al., 2016; Alvarado and Schwartz, 2017). The pathogenesis of ZIKV has not yet been fully investigated, and in this review, we discuss the possible mechanisms involved in pathogenesis. Currently, no specific antiviral drug is available to treat ZIKV infection, but there are several promising drug targets encoded by the virus itself or present in the host cells. ZIKV’s structural protein E and non-structural proteins, such as NS2B–NS3, NS3, and NS5, as well as host proteins involved in the ZIKV replication can serve as targets for the development of anti-ZIKV therapeutics.

## Pathogenesis of ZIKV Infection

The high neurological effects of ZIKV affect fetal brain development resulting in microcephaly, cortical malformation, and intracranial calcification (Chavali et al., 2017). There may be a correlation between ZIKV infection and Guillain–Barré syndrome (GBS), but the mechanisms are still unclear. The outbreak of the ZIKV epidemic in the past 5 years confirms the relationship between ZIKV infection and microcephaly or other neurological diseases during pregnancy (Lin et al., 2018; Vue and Tang, 2021). ZIKV infection can result in its persistence within different body fluids including semen, saliva, tears, and urine as well as in target organs including immune isolation sites such as the eyes, brain, and testes. Infectious virions as well as viral nucleic acids were also detected in the female reproductive tract after ZIKV infection (Proenca-Modena et al., 2018).

In a study related to microcephaly, ZIKV was demonstrated to trigger endoplasmic reticulum (ER) stress and unfolded protein response (UPR) in the cerebral cortex of human fetuses and in cultured human neural stem cells. ZIKV selectively interferes with the development of the cerebral cortex. ZIKV-infected progenitor cells produce lower number of neuron projections that eventually precipitate in the cerebral cortex. Moreover, sustained ER stimulation leads to apoptosis. The use of pharmacological inhibitors demonstrated that UPRs can counteract these pathophysiological mechanisms to prevent microcephaly in ZIKV-infected mouse embryos (Chavali et al., 2017; Gladwyn-Ng et al., 2018). ZIKV also impairs neurogenic balance through non-cellular autonomic mechanisms. Vertical transmission of the virus, targeting apical progenitors inducing ER stress and activation of the UPR, can ultimately lead to microcephaly, and the damage to the physiological UPR seems to be the main mechanism of action (Gladwyn-Ng et al., 2018). It has been reported that a single S139N mutation in the prM protein can contribute to ZIKV infectivity in both human and mouse neural progenitor cells, which resulted in more severe microcephaly and higher mortality in mice (Yuan et al., 2017). Interestingly, TLR3 stimulation was shown to activate multiple genetic regions as well as regulate the occurrence of axons in neural progenitors, cell proliferation, and anti-apoptotic processes, which may result in a ZIKV-mediated microcephaly phenotype (Kong et al., 2018). It has been demonstrated that NS2A destroys the development of mammalian cortical nerves and reduces the proliferation of radial glial cells by degrading the adhesion-binding complex (Kong et al., 2018). On the other hand, NS4A and NS4B of the mutant ZIKV support viral replication by synergistically inhibiting the Akt–mTOR pathway leading to cellular dysregulation and promotion of autophagy (Kong et al., 2018). It is of interest to note that ATG16 (autophagy-associated protein) greatly affects placental susceptibility and vertical transmission of ZIKV (Chiramel and Best, 2018). ZIKV replication also blocks the stress response pathway by inhibiting stress granule proteins, in which NS3 and NS4A play key roles (Miner and Diamond, 2017). NS4A impairs the interaction of RIG-I (retinoic acid-inducible gene I)-like receptor (RLR)–mitochondria-antiviral-signaling protein (MAVS), followed by an induced antiviral immune response. Importantly, NS4A inhibits type I IFN induced by RIG-I and a melanoma differentiation-associated protein 5 (MDA5). The RLR pathway is required for the induction of type I IFN responses to ZIKV infection in human trophoblast cells (Ma et al., 2018). It has been reported that NS4A mediates cell hypertrophy and delay in growth through the rapamycin-mediated cellular stress response pathway (Kong et al., 2018). It was found that mice lacking IFN-α/β signaling expressed high levels of ZIKV in the serum, spleen, brain, spinal cord, and testis indicating that IFN-α/β signaling plays a role in limiting ZIKV infection (Miner and Diamond, 2017). On the other hand, NS5 expression causes IFN-regulated proteasome degradation of the transcriptional activator STAT2, which also reflects the importance of STAT2 during ZIKV infection. Interestingly, ZIKV IFN antagonism may have species restriction because STAT2 levels were not affected in ZIKV-infected primary mouse embryonic fibroblasts although impaired ZIKV replication was observed (Olagnier et al., 2016). Taken together, ZIKV utilizes non-structural proteins to block both the pathways of type I IFN and mTOR signaling, which normally inhibit viral replication and neurogenesis and induce cell growth arrest.

Replication of ZIKV results in a delay in mitochondrial apoptosis in human epithelial cells. In addition, studies have shown that ZIKV can infect and replicate in mouse neurons and astrocytes. ZIKV can damage human brain cells by reducing the vitality and growth of neurospheres and brain structures (Miner and Diamond, 2017), but the mechanism is still unclear. It has been found that silencing of the m6A encoder reduces the synthesis of ZIKV and the release of virions from virus-producing cells, indicating that the host RNA methyltransferase mechanism can act as a negative post-transcriptional regulator (Kong et al., 2018). P53 activation of human pluripotent stem cells (hPSCs) after ZIKV infection was observed, and a small number of P53 effector proteins also play a key role in ZIKV infection (Kong et al., 2018).

ZIKV infection was shown to preferentially infect hPSC-derived neural progenitor cells causing cell death and mitotic damage in mice, a plausible potential mechanism for the development of microcephaly (Onorati et al., 2016). *In-vitro* ZIKV infection of human neutrosphere organ culture impairs the growth of cells and also increases cell death (Miner and Diamond, 2017). Inhibitors of TBK1 (phosphorylated TANK-binding kinase 1) block mitosis, stimulate excess centrosomes in neuroepithelial stem (NES) cells, and exacerbate ZIKV-associated NES cell death (Onorati et al., 2016). In addition, infection of cranial neural crest cells leads to the production of inflammatory cytokines that promote apoptosis of neural progenitor cells. Therefore, direct infection of neural progenitor cells may not be the only factor leading to microcephaly (Miner and Diamond, 2017). A recent study demonstrated that ZIKV has increased monocyte adhesion and transmigration, which favored ZIKV transmission to neural cells (Ayala-Nunez et al., 2019).

TLR3 was identified as an initial immune receptor involved in ZIKV infection in human fibroblasts leading to type I and type II IFN responses. ZIKV can also infect immature Mo-DCs by interacting with dendritic cell-specific intercellular adhesion molecule-3-grabbing non-integrin (DC-SIGN), also known as CD209, which is a C-type lectin receptor present on the surface of both macrophages and dendritic cells. In the absence of DC-SIGN, the phosphatidylserine receptors of the TIM and TAM families may act as receptors or attachment factors for the virus. For example, in skin fibroblasts and epidermal keratinocytes lacking DC-SIGN, the TAM receptor AXL induces the entry of ZIKV. Since high levels of AXL receptors are present on the radial glial cells (Olagnier et al., 2016), AXL may play an important role in the entry of ZIKV into nerve cells (Olagnier et al., 2016). Dermal fibroblasts and epidermal keratinocytes are also targets of ZIKV infection and aid in the transmission of infection to Langerhans cells (Olagnier et al., 2016). For flaviviruses, effective antagonism of the innate antiviral response is critical for viremia-dependent maintenance of the vector–host cycle (Miner and Diamond, 2017). Heat shock proteins (HSPs) were found to be a major factor in the pathogenesis of many viruses, and HSP70 was shown to play an important role in influencing ZIKV entry, replication and release, and infection of host cells (Pujhari et al., 2019).

MicroRNAs (miRNAs) are involved in the regulation of a wide variety of cellular and physiological processes. Researchers examined 599 miRNAs and 770 mRNAs in ZIKV-infected primary mouse neurons by using the nCounter technology. They found that ZIKV infection could cause global downregulation of miRNAs, with an inverse correlation of the target host mRNAs. Moreover, downregulation of miRNA caused upregulation of neuroinflammation and apoptotic genes (Azouz et al., 2019), demonstrating that ZIKV-modulated miRNAs and mRNAs regulate the pathways related to neurological development and neuroinflammatory responses.

## The Development of Anti-ZIKV Therapeutics

Approaches targeting either ZIKV proteins (e.g., NS2B–NS3, NS3, NS5, and E proteins) or host proteins (e.g., glucose transporter, HSP70, dihydrofolate reductase, cellular kinase) have been applied for the development of anti-ZIKV therapeutics (**Table 1**).

**Table 1:** Summary of ZIKV inhibitors targeting different viral and host proteins.

Inhibitor	Target	IC_50_ or EC_50_ (μM)	Development stage	Reference
Inhibitors targeting viral proteins				
Polyanion suramin	NS2B–NS3	IC_50_ = 47 μM	Approved antiparasitic drug with antiviral properties	Coronado et al. (2018)
Bromocriptine	NS2B–NS3	IC_50_ = 13.04 ± 2.00 μM	Preclinical study	Chan et al. (2017)
Novobiocin	NS2B–NS3	In the CPE inhibition assay: IC_50_ = 86.92 μM; in the plaque reduction assay: IC_50_ = 24.82 μM	Also known as albamycin or cathomycin, which is a clinically used antibiotic	Yuan et al. (2017)
Compounds 1 and 2	NS3	IC_50_ = 8.5 and 15.2 μM	Preclinical study	Zhu et al. (2019)
Asunaprevir and simeprevir	NS3	EC_50_ = 4.7 µM and 0.4 µM	FDA-approved	Pathak et al. (2020)
Sofosbuvir	NS5-RdRp	Not tested	FDA-approved for the treatment of HCV infection	Ferreira et al. (2017)
4-HPR	NS5	In the plaque reduction assay: EC_50_ = 2.6 ± 0.3 μM; in the RT-qPCR: EC_50_ = 3.2 ± 1.7 μM	Preclinical study	Wang et al. (2017)
3-110-22	E protein	IC_90_ = 4.2 ± 0.2 μM	Preclinical study	Li et al. (2019)
ZINC40621658	E protein	Not tested	Preclinical study	Fernando and Fernando (2017)
Atovaquone	E protein	IC_90_ = 2.1 μM	FDA-approved for the treatment of malaria	Yamamoto et al. (2020)
Inhibitors targeting host proteins				
Phloretin	Glucose transporter	EC_50_ = 22.85 µM [MR766 (African genotype)] and 9.31 µM [PRVABC59 (Puerto Rico genotype)]	Preclinical study	Lin et al. (2019)
MTX	DHFR	IC_50_ = 0.245 µM (Vero cells) and 0.334 µM (hNSCs)	Clinically used as an anticancer chemotherapy and antirheumatoid agent	Beck et al. (2019)
JG40, JG132, and JG345	HSP70	Not tested	Preclinical study	Taguwa et al. (2019)
QC, MQ, and GSK369796	Autophagy pathway	EC_50_ = 2.27 ± 0.14 μM (QC); EC_50_ = 3.95 ± 0.21 μM (MQ); EC_50_ = 2.51 ± 0.09 μM (GSK369796)	FDA-approved antimalarial drugs	Balasubramanian et al. (2017)
Memantine	NMDA	Not tested	Approved by international regulatory agencies for the treatment of Alzheimer’s disease	Costa et al. (2017)
AR-12	Cellular kinase	IC_50_ < 2 μM	Phase I clinical trials as an anticancer agent in adult patients with advanced or recurrent solid tumors or lymphomas (Clinical Trials registration no. NCT00978523)	Chan et al. (2018)
MMPD	IMPDH	EC_50_ = 0.6 μM	FDA-approved for the treatment of HCV infection	Tong et al. (2018)

Abbreviations: HCV, hepatitis C virus; 4-HPR, N-(4-hydroxyphenyl) retinamide; MTX, methotrexate; DHFR, dihydrofolate reductase; HSP70, heat shock protein 70; QC: quinacrine; MQ, mefloquine; hNSCs, human neural stem cells; RdRp, RNA-dependent RNA polymerase; NMDA, N-methyl-D-aspartate; MMPD, merimepodib; IMPDH: inosine-5′-monophosphate dehydrogenase.

### NS2B–NS3 Protease Inhibitors

The NS2B–NS3 protease of ZIKV plays a crucial role in the processing of the viral precursor polyprotein during viral replication, and acquisition of the crystal structures of the unlinked NS2B–NS3 protease from ZIKV accelerates the discovery of structure-based antiviral drugs (Zhang et al., 2016; Voss and Nitsche, 2020). Since the epidemic ZIKV outbreak in the Americas, several inhibitors of this protease have been reported. Polyanion suramin, an approved antiparasitic drug with antiviral properties, inhibits the ZIKV complex NS2B–NS3 proteinase with an IC_50_ value of 47 μM. Suramin blocked the ser135 residue, which interacts with the catalytically active histidine residue as determined by saturation transfer difference NMR spectroscopy, molecular docking, and molecular dynamics studies (Coronado et al., 2018). After testing 71 HCV NS3/NS4 inhibitors, Lee et al. identified a non-peptide small-molecule competitive inhibitor, which stabilized the closed conformation of ZIKV protease with an IC_50_ and dissociation constant (*K*_D_) of 5–10 μM (Lee et al., 2017). Bromocriptine inhibited ZIKV replication by interacting with several active site residues of the proteolytic cavity involving H51 and S135 in the ZIKV NS2B–NS3 protease complex, and it might occupy the active site of the enzyme (Chan et al., 2017). Novobiocin and lopinavir–ritonavir were verified to inhibit virus replication *in vitro* through repurposing clinically approved drugs that inhibited the key enzymes in large chemical library screening experiments. Binding between novobiocin and ZIKV NS2B–NS3 was found to be highly stable as determined by molecular docking and molecular dynamics simulation experiments (Yuan et al., 2017). Three potent candidates, temoporfin, niclosamide, and nitazoxanide, targeting flavivirus NS2B–NS3 interactions with nanomolar potencies have been identified by a split luciferase complementation-based high-throughput screening assay from a total of 2,816 approved and investigational drugs. Moreover, the most potent compound, temoporfin, not only inhibited ZIKV replication in human placental and neural progenitor cells but also prevented ZIKV-induced viremia and mortality in immunocompetent BALB/c and A129 mouse models, which makes it a promising candidate for treating ZIKV infection (Li et al., 2017). An in-house database of 150 natural and semisynthetic compounds against ZIKV NS2B–NS3 protease has been screened using docking-based virtual screening, and a promising hit, pedalitin, was demonstrated to inhibit the protease with an IC_50_ value of 5 μM and to show significant activity at 250 and 500 μM, but with slight toxicity in Vero cells. So, studies toward the development of pedalitin to treat ZIKV infection need to be further optimized (Lima et al., 2021).

### NS3 Protease Inhibitors

NS3 encodes a serine protease, RNA helicase, nucleoside triphosphatase, and RNA triphosphatase. Therefore, it is an attractive target for an antiviral drug. For computational drug discovery, structure-based virtual screening using PyRx 0.8 software is an efficient way of filtering compounds. Four US FDA-approved drugs against dengue virus (DENV), namely, amodiaquine, prochlorperazine, quinacrine, and berberine, were used as ligands for molecular docking analysis against the NS3 protein of ZIKV. The results indicated that berberine exhibited the highest binding affinity of −5.8 kcal/mol. Based on the properties of berberine, more potential compounds were identified (Sahoo et al., 2016), suggesting that the screening platform is of importance for developing inhibitors against ZIKV infection. Based on ZIKV NS3 helicase, the National Cancer Institute (NCI) identified two novel lead compounds (named compounds 1 and 2) from the screening of almost 250,000 compounds using a molecular docking approach. Compounds 1 and 2 inhibited ZIKV infection with IC_50_ values at the micromolar level (8.5 and 15.2 μM) and interacted with ZIKV NS3 helicase as shown by the structure–activity relationship studies. Moreover, compound 1 had a higher affinity (−14.09 kcal/mol) to NS3 helicase than compound 2 (−8.05 kcal/mol) (Zhu et al., 2019). A pharmacophore anchor (PA) model was constructed aiming at ZIKV NS3 protease, and two FDA drugs, asunaprevir and simeprevir, showed potent anti-ZIKV activities with EC_50_ values 4.7 and 0.4 µM, suggesting that these two FDA-approved drugs are promising for anti-ZIKV treatment (Pathak et al., 2020).

### NS5 Protease Inhibitors

NS5 encodes methyltransferase and RNA-dependent RNA polymerase (RdRp) at its N-terminus and C-terminus. Methyltransferase is crucial in viral RNA genome capping (Chambers et al., 1990; Choi and Rossmann, 2009), replication, and immune evasion (Dong et al., 2014). Furthermore, it may also play a role in modulating the activity of the cellular spliceosome (Diamond, 2009; Best, 2017). The structure of NS5 is highly conserved having a GTP/Cap-binding pocket and SAM-binding pocket (Best, 2017). Researchers discovered MTase inhibitors using molecular modeling to identify the additional hydrophobic region present within the structure of ZIKV MTase, which is uniquely conserved among the flavivirus Mtases (Stephen et al., 2016). Small nucleoside inhibitors aim to inhibit the activity of ZIKV NS5-RdRp, which could lead to chain termination or lethal replication (Ranjith-Kumar et al., 2002; Boldescu et al., 2017; Garcia et al., 2017). Sofosbuvir, a class B FDA-approved drug, induces an increase in A to G mutations in the viral genome and inhibits the replication of ZIKV in a cell lineage in a dose-dependent manner. The residues which sofosbuvir targets are important for RdRp of an African ZIKV strain from the 1950s (Sacramento et al., 2017). In a mouse model, all the ZIKV-infected mice without any treatment died. However, 40% of sofosbuvir-treated ZIKV-infected animals survived (Ferreira et al., 2017). N-(4-hydroxyphenyl) retinamide (4-HPR), which targets DENV NS5, can block the interaction between ZIKV NS5 and the host cell importin (IMP) α/β1 heterodimer at low micromolar concentrations (Wang et al., 2017). One recent study adopted a strategy to explore the lead compounds from *Azadirachta indica* against ZIKV *via* concurrent inhibition of the NS2B–NS3 protease and NS5 proteins using molecular simulations. Structure-based virtual screening of 44 bioflavonoids reported in *A. indica* against the crystal structures of targeted ZIKV proteins resulted in the identification of the top four common bioflavonoids, serving as promising therapeutic inhibitors of NS2B–NS3 protease and NS5 proteins against ZIKV for further experimental assessment (Kumar et al., 2022).

### E Protein Inhibitors

E protein attaches to the receptors on the host cell membrane. The virus is internalized *via* endocytosis mediated by a clathrin protein in a low pH environment, suggesting that E protein is also an attractive inhibitor target. Peptides (P5) from the Japanese encephalitis virus (JEV) E protein stem domain (from A424 to F445) blocked ZIKV infection with an IC_50_ value at the micromolar level and also reduced the infection in the brain of type I and II IFN receptor-deficient AG6 mice (Chen et al., 2017). The small molecule E inhibitors like 3-110-22, which target a pocket located at the interface of domains I and II, blocked the entry of multiple flaviviruses *in vivo* (Li et al., 2019). The small molecular probes revealed that ZVEP is druggable in the cryo-EM structure of ZIKV (5IRE), where four ligands bind to the glycan-binding domain constituted by residues VAL153 and ASN154. ZINC40621658 is possibly a potent inhibitor, which acts *via* allosteric blockage of the glycan-binding domain (Fernando and Fernando, 2017). An *in-vitro* quantitative mosquito-cell-based membrane-fusion assay was developed to screen an FDA-approved drug library for a potential membrane fusion inhibitor using ZIKV E protein, and an antimalarial agent called atovaquone was identified. It was demonstrated that atovaquone could block not only the ZIKV infection of mammalian cells with an IC_90_ value of 2.1 μM but also four distinct serotypes of DENV with IC_90_ values of 1.6–2.5 μM, suggesting atovaquone as a potential candidate to treat illnesses caused by ZIKV as well as DENV (Yamamoto et al., 2020).

### Anti-ZIKV Agents Targeting Host Proteins

Alpha2,3-linked sialic acid facilitates the internalization of ZIKV (Tan et al., 2019). For the propagation of ZIKV, the glucose utilization pathway is important. Phloretin, a glucose transporter inhibitor, disrupts cellular glucose availability by using 2-deoxy-D-glucose, which cannot further proceed to glycolysis (Lin et al., 2019). Methotrexate (MTX) inhibits dihydrofolate reductase (DHFR) and can act against ZIKV infection (Beck et al., 2019). Drugs targeting host proteases are effective as antivirals at the dose of reducing replication without appreciable toxicity to the host cells. In human placental cells but not in Vero cells, ZIKV requires intracellular glutathione for replication, and intracellular glutathione acts as an active defense against viral infections. The formulation of a specific free-form amino acid (FFAAP) comprising cysteine, glycine, and a glutamate source, along with a minute concentration of selenium, inhibited ZIKV replication with ED_90_ values of 2.5 μM in human cells and 4 mM in Vero cells by blocking intracellular biosynthesis of glutathione (Vasireddi et al., 2019).

Autophagy, a cellular catabolic pathway delivering cytoplasmic cargo to the lysosome for degradation, is a fundamental process in ZIKV infection. The Akt–mTOR signaling pathway causes aberrant activation of autophagy, viral replication, and propagation, which is inhibited by ZIKV (Gratton et al., 2019). Three forms of autophagy (microautophagy, chaperone-mediated autophagy, and macroautophagy) are identified so far (Levine and Kroemer, 2008). There are autophagy-inhibiting lysosomotropic agents, such as quinacrine (QC), mefloquine (MQ), and GSK369796. QC, MQ, and GSK369796 showed antiviral activity against ZIKV with EC_50_ values of 2.27 ± 0.14, 3.95 ± 0.21, and 2.51 ± 0.09 μM, respectively, by inhibiting the later stages of autophagy (Balasubramanian et al., 2017).

Blocking N-methyl-D-aspartate (NMDA) rescues ZIKV-induced neurodegeneration using an NMDA receptor antagonist, memantine. The death of neuronal cells was reduced at 10 μM and totally prevented at 100 μM of memantine (Costa et al., 2017). AR-12, a celecoxib derivative and a cellular kinase inhibitor, inhibited ZIKV strains belonging to both the African and Asian/American lineages in Huh-7 and neuronal cells with a consistent IC50 value <2 μM. A192 mice treated with AR-12 showed higher survival rate, less body weight loss, and lower ZIKV RNA loads in the blood and tissues (Chan et al., 2018). Researchers found that merimepodib (MMPD, VX-497), a potent inhibitor of inosine-5′-monophosphate dehydrogenase (IMPDH), inhibited ZIKV RNA replication with an EC_50_ value of 0.6 μM, reduced the production of ZIKV, and also enhanced the suppression of virus production in combination with ribavirin as well as favipiravir (Tong et al., 2018). Giovannoni’s team found that ZIKV infection triggers aryl hydrocarbon receptor (AHR) activation by using genome-wide transcriptional studies and AHR inhibition with a nanoparticle-delivered AHR antagonist or an inhibitor developed for human use suppressed ZIKV replication and ameliorated newborn microcephaly in pregnant SJL mice through increasing IFN-I signaling (Giovannoni et al., 2020).

## Conclusions and Perspectives

As a mosquito-borne positive-stranded RNA virus, ZIKV caused a large-scale infection in 2015, and it is now causing an unprecedented outbreak in the Americans. Although ZIKV infection generally causes no symptoms or only a mild self-limiting disease, it has been confirmed to be related to some severe neurological diseases such as microcephaly and GBS. ZIKV poses a serious threat to public health around the world; thus, it highlights the necessity to develop safe and effective vaccines and antiviral drugs to fight against ZIKV infection. Approaches directing both proteins of ZIKV and different host proteases have been developed for anti-ZIKV agents. ZIKV proteases such as NS2B–NS3, NS5, and E proteins are relative ideal targets. Host proteins such as glucose transporter, DHFR, and cellular kinase are appropriate targets for anti-ZIKV designation. Apart from the targets mentioned in this review, more novel targets might need to be developed for promising therapeutics against ZIKV infection. This review may provide guidance for further development of effective therapeutics against ZIKV infection.

## Author Contributions

KL and QJ contributed to the preparation of the manuscript draft. SJ and NZ conceived the topic of this review and revised the manuscript. All authors contributed to the article and approved the submitted version.

## Funding

This work was supported by the Scientific Research Foundation of Zhejiang University City College (Grant No. J-202106) and Hangzhou Science Technology Bureau to NZ.

## Conflict of Interest

The authors declare that the research was conducted in the absence of any commercial or financial relationships that could be construed as a potential conflict of interest.

## Publisher’s Note

All claims expressed in this article are solely those of the authors and do not necessarily represent those of their affiliated organizations, or those of the publisher, the editors and the reviewers. Any product that may be evaluated in this article, or claim that may be made by its manufacturer, is not guaranteed or endorsed by the publisher.
