# Corrigendum: Advancement in the development of therapeutics against Zika virus infection

**DOI:** 10.3389/fcimb.2022.1006226

**Published:** 2022-08-26

**Authors:** Kangchen Li, Qianting Ji, Shibo Jiang, Naru Zhang

**Affiliations:** ^1^ Department of Clinical Medicine, School of Medicine, Zhejiang University City College, Hangzhou, China; ^2^ Key Laboratory of Medical Molecular Virology of Ministry of Education (MOE), National Health Commission (NHC) and Chinese Academy of Medical Sciences (CAMS), School of Basic Medical Sciences and Shanghai Institute of Infectious Disease and Biosecurity, Fudan University, Shanghai, China

**Keywords:** Zika virus, pathogenesis, therapeutics, NS2B–NS3 protease inhibitors, NS3 protease inhibitors, NS5 protein inhibitors, E protein inhibitors, host protein inhibitors

## Text Correction

In the published article, there was an error. [There is a spelling mistake by writing “**NS5 protease inhibitors**” in key words on page 1 and in one of the subheadings named “**NS5 Protease Inhibitors**” on page 4].

A correction has been made to **[“Key words” and “THE DEVELOPMENT OF ANTI-ZIKV THERAPEUTICS”]**, *[NS5 Protease Inhibitors*], [the subheading of “*NS5 Protease Inhibitors”*]. This sentence previously stated: “[NS5 Protease Inhibitors]”

The corrected sentence appears below: “[NS5 Protein Inhibitors]”

The authors apologize for this error and state that this does not change the scientific conclusions of the article in any way.

## Publisher’s note

All claims expressed in this article are solely those of the authors and do not necessarily represent those of their affiliated organizations, or those of the publisher, the editors and the reviewers. Any product that may be evaluated in this article, or claim that may be made by its manufacturer, is not guaranteed or endorsed by the publisher.

